# Biomedical Applications of Metallosupramolecular Assemblies—Structural Aspects of the Anticancer Activity

**DOI:** 10.3389/fchem.2018.00620

**Published:** 2018-12-14

**Authors:** Anife Ahmedova

**Affiliations:** Laboratory of Biocoordination and Bioanalytical Chemistry, Faculty of Chemistry and Pharmacy, Sofia University, Sofia, Bulgaria

**Keywords:** metallacycle, metallacage, anticancer, capsule, encapsulation

## Abstract

The design and development of metallosupramolecular systems has resulted in construction of a myriad of fascinating structures with highly diverse properties and potential applications. Assessment of the biomedical applications of metallosupramolecular assemblies is an emerging field of research that stems from the recently demonstrated promising results on such systems. After the pioneering works of Therrien and coworkers on organometallic Ru-cages with promising anticancer properties, this topic has evolved to the more recent studies on bioactivity of supramolecular coordination complexes built from different metal ions and various multidentate ligands. Sufficient amount of data on the anticancer activity of metallosupramolecules has already been reported and allows outlining some general tendencies in the structural aspects of the biological activity. The main structural properties of the complexes that can be readily modified to enhance their activity are the size, the shape and charge of the formed complexes. Moreover, the intrinsic properties of the building components could predetermine some of the main characteristics of the overall supramolecular complex, such as its optical properties, chemical reactivity, solubility, etc., and could, thereby, define the areas of its biomedical applications. The unique structural property of most of the metallosupramolecular assemblies, however, is the presence of a discrete cavity that renders a whole range of additional applications resulting from specific host-guest interactions. The encapsulations of small bioactive or fluorescent molecules have been employed for delivery or recognition purposes in many examples. On the other hand, metallosupramolecules have been imbedded into target-specific polymeric nanoparticles that resulted in a successful combination of their therapeutic and diagnostic properties, making them promising for theranostic application in cancer treatment. The aim of this review paper is to mark out some key tendencies in the reported metallosupramolecular structures in relation with their biological activity and potential areas of biomedical application. In this way, a useful set of guidelines can be delineated to help synthetic chemists broaden the application areas of their supramolecular systems by few structural changes.

## Introduction

The coordination bond directed self-assembly processes have been broadly exploited in building metallosupramolecular species with diverse composition (Bilbeisi et al., 2014), discrete size and shape (Schmidt et al., 2014; Cook and Stang, 2015), and variety of useful properties. Unarguably, the aesthetics of these highly symmetric metallosupramolecules has been the initial source of inspiration for many chemists. This resulted in not only building a myriad of fascinating structures but also in establishing the main principles in the design and synthesis of metallosupramolecular complexes with predefined shapes by exploiting the bond directionality and the coordination properties of the metal ions, and intelligent design of the linking organic ligands (Datta et al., 2018). Nowadays, the main targets are the specific properties of the metallosupramolecular complexes in view of their practical applications that range from chemical catalysis to multi-purpose and stimuli-responsive advanced materials (McConnell et al., 2015). The demonstrated high potential to purposefully combine the properties of metal ions with that of organic ligands has recently attracted the attention of bioinorganic chemists which added up more facets to the range of practical use of the metallosupramolecules—their biological application for therapy and/or diagnostics (Casini et al., 2017; Leung et al., 2017).

The continuous studies related to the clinical use of Pt-complexes as anticancer drugs have evolved to establishing structure-activity relationships for this class of compounds. It is, therefore, not surprising that the first studies on the potential biological applications of metallosupramolecules had focused on anticancer activity of Pt-linked metallosupramolecular cycles (Mounir et al., 2007) and interaction of Fe- or Ru-linked metallohelicates with DNA fragments (Hotze et al., 2006; Malina et al., 2008). This had also given impetus to explore a broader range of metal complexes including the organometallic half-sandwich structures (Aird et al., 2002). Some of the initial examples for studying in details the anticancer activity profiles of coordination driven self-assemblies are the organometallic Ru(II) metallacycles and cages that have been extensively investigated (Therrien et al., 2008; Smith and Therrien, 2011; Dubey et al., 2013; Vajpayee et al., 2013). Thereby, the topic of metal-based drugs had naturally reached for the advantages that metallosupramolecular entities can provide—the major one is their encapsulation and delivery potential in addition to the intrinsic properties of their building components (Therrien et al., 2008; Schmitt et al., 2012; Therrien, 2013; Ma and Zhao, 2015).

As a multidisciplinary field, the topic of biomedical application of metallosupramolecules requires mutual understanding between synthetic chemists and biologists with the continuing help of specialist in photochemistry, pharmacology, bioimaging, etc. With the ultimate goal to facilitate this process, we intended to describe the recent achievements in the design of metallacycles, metallacages and helicates, and metallosupramolecular capsules and barrels in view of their anticancer properties, recognition of biomolecules with therapeutic implication, as well as improved delivery of bioactive molecules for therapeutic or imaging purposes. The examples of advanced nanomaterials that are based on, or incorporate, metallosupramolecular entities have grown in number and enriched in functionality, and are, therefore, given a major importance in this review paper. While the specific areas of biological applications of the metal-based supramolecules are described, the effect of their characteristic structural features is emphasized. This includes the charge, the shape and the size of the metallosupramolecules, as well as their specific cavity, which reflect the overall stability, reactivity, encapsulation potential, etc.—all being related to the biologically relevant applications. Ultimately, the wide variety of structures with desired composition and properties that can be suitably obtained through supramolecular coordination chemistry, makes this class of compounds a generous area for exploration not only from synthetic point of view but also in view of their biological activity.

## Metallacycles

As two-dimensional structures, the properties of metallacycles can be tuned by mere combination of the intrinsic characteristics of the metal ion and the linking organic ligands, thus forming supramolecular entities of variable size, shape, and charge. The stability issues have been overcome by employing inert metal ions as metal hinges or organometallic clips that form more robust metal-carbon bonds. Among the latter are the half-sandwich Ru(II) arene clips with bridging oxalate ligands that have been developed by Süss-Fink and coworkers and employed in the construction of the first organometallic Ru(II) metallamacrocycles (Yan et al., 1997). As the anticancer properties of ruthenium complexes came into focus after their introduction into clinical trials, and the first reports on the *in vitro* and *in vivo* cytotoxicity of a series of novel Ru(II) organometallic arene complexes (Aird et al., 2002), the topic expanded further on to the anticancer potency of metallosupramolecular assemblies with the pioneering works of Therrien and coworkers (Therrien et al., 2008; Mattsson et al., 2009). Several groups had greatly contributed in the field of biological applications of metallacycles of different shape and composition. The anticancer properties of series of tetracationic metallacycles, formed by [2+2] assembly between dipyridyl ligands and Ru(II) arene clips with oxalate or dioxydo-1,4-quinonato types of bridges of different size, have been thoroughly studied and later reviewed by Cook, Stang and coworkers (Cook et al., 2013). While this class of organometallic Ru(II) metallacycles demonstrated promising cytotoxicity profiles and structure-activity relationships could be outlined, the bis-ruthenium arene clips played further role in building 3D supramolecular cages and boxes by coordinating with tripodal or tetrapodal pyridyl ligands. Thereby, additional functionality has been introduced in terms of guest-encapsulation capability that further empowered the biological potential of the organometallic arene-metallosupramolecules. This topic has been extensively reviewed (Zhang et al., 2017) and therefore shall be left outside the scope of this paper.

**Figure 1:**
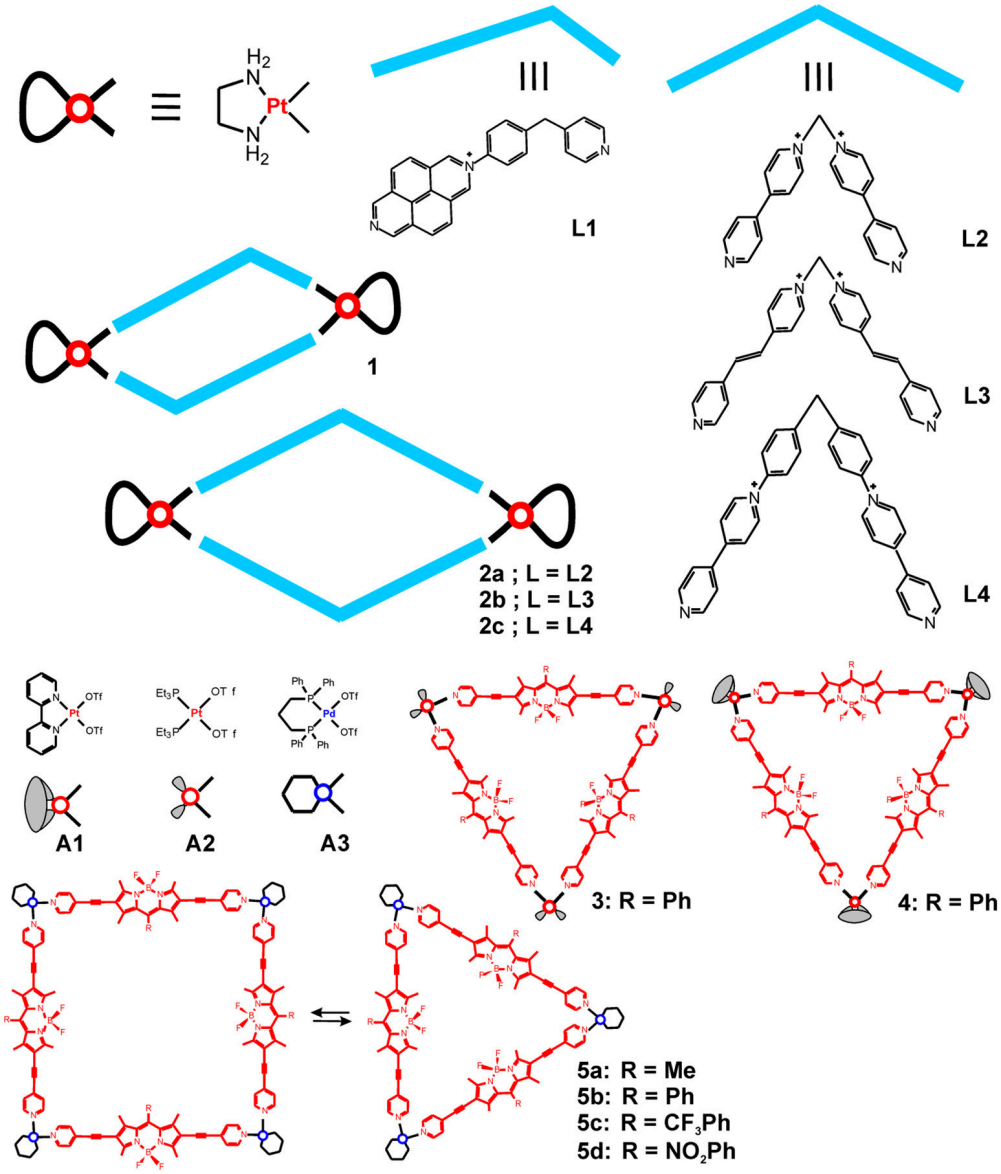
Metallosupramolecular rhomboids and triangles based on cis-blocked Pt(II) and Pd(II) corners and bent shaped or linear donors.

Pt(II) and Pd(II)-cornered square-planar complexes have long been utilized in building highly symmetric 2D and 3D structures of desired shape and size. As chemically more inert units, the ethylenediamine-Pt(II) corners have been used to form [2+2] self-assemblies with rhomboidal structures by coordinating with bent-shape bipyridyl ligands. Some recent examples utilized the cationic N-monoalkylated 4,4′-bipyridinium or 2,7- diazapyrenium based ligands (Terenzi et al., 2012; Domarco et al., 2017) depicted in Figure 1. Similarly to earlier reports on DNA-binding capability of tetranuclear Pt(II) squares of linear bipyridyne ligands (Mounir et al., 2007), the formed hexa- and octacationic Pt(II) rectangular metallacycles strongly bind native and G-quadruplex DNA. While the smaller size, unsymmetrical 2,7- diazapyrenium ligand is able to intercalate the DNA, the formed metallacycle (**1**) binds DNA ten times more strongly and bends its structure, thereby blocking the DNA transaction that possibly reflects on the observed anticancer activity against four types of human cancer cell lines (Terenzi et al., 2012). Similarly, the octacationic Pt(II)-cornered metallacycles **2a-c** exhibit moderate anticancer activity as compared to cisplatin (Table 1), which has also been related to their strong binding to DNA (Domarco et al., 2017). Interestingly, the size of the rectangular box determines their effect on the DNA binding and the G4 folding process—the smaller Pt-box exhibited lower binding affinity but better selectivity for a particular G4 topology. The middle-sized box **2b** showed highest anticancer activity against the tested cancer cells, including the cisplatin resistant cell line VM-1, which, however, can be associated with the detected moderate toxicity of the free ligand (**L3**) against the U2OS cells (38.2 ± 3.4 μM).

**Table 1:** Cytotoxicity data of selected metallacycles against a panel of human cancer cells.

**Compound**	**IC_**50**_ (hrs of incubation)/Type of cells (IC_**50**_ of ref. compound)**	**IC_**50**_ (hrs of incubation)/Type of cells (IC_**50**_ of ref. compound)**	**IC_**50**_ (hrs of incubation)/Type of cells (IC_**50**_ of ref. compound)**	**IC_**50**_ (hrs of incubation)/Type of cells (IC_**50**_ of ref. compound)**	**References**
**1**	19.24 ± 7.13 μM (96 h)/SISO (0.24 ± 0.05/CDDP)	3.12 ± 1.22 μM (96 h)/A-427 (1.27 ± 0.25/CDDP)	9.47 ± 3.12 μM (96 h)/LCLC-103H (1.09 ± 0.40/CDDP)	7.61 ± 6.65 μM (96 h)/5637 (0.37 ± 0.08/CDDP)	Microtitre assay (Terenzi et al., 2012)
**2a**	37.8 ± 3.3 μM (72 h)/U2OS (3.8 ± 3.0/CDDP)	>50 μM (72 h)/VM-1 (4.43 ± 0.05/CDDP)	32.1 ± 7.4 μM (72 h)/MCF7 (6.7 ± 0.3/CDDP)		Domarco et al., 2017
**2b**	28.9 ± 4.1 μM (72 h)/U2OS (3.8 ± 3.0/CDDP)	31.8 ± 0.5 μM (72 h)/VM-1 (4.43 ± 0.05/CDDP)	39.4 ± 3.4 μM (72 h)/MCF7 (6.7 ± 0.3/CDDP)		Domarco et al., 2017
**2c**	42.3 ± 4.6 μM (72 h)/U2OS (3.8 ± 3.0/CDDP)	>50 μM (72 h)/VM-1 (4.43 ± 0.05/CDDP)	39.6 ± 4.5 μM (72 h)/MCF7 (6.7 ± 0.3/CDDP)		Domarco et al., 2017

More recently, new types of cornered Pt(II) and Pd(II) square-planar complexes (**A1-A3**, Figure 1) have been utilized in building metallosupramolecules with potential biomedical use. Notable examples are the highly fluorescent triangles constructed from boron dipyrromethene (BODIPY) based dipodal ligands and Pt(II) or Pd(II) corners, as they combine cellular imaging and cancer treatment potency of the building components. The highly emissive BODIPY-Platinum supramolecular triangles (**3** and **4** in Figure 1) proved potent for intracellular visualization by confocal microscopy as well as for photodynamic therapy (PDT) and chemotherapy (Zhou et al., 2018a). Indeed, these metallacycles were used in the form of nanoparticles (NPs) that have been prepared through nanoprecipitation. More favorable cellular internalization of the NPs, formed from the cationic metallacycles **3** and **4**, has been observed in comparison with the free, pyridine functionalized BODIPY ligand. Furthermore, metallacycles **3** and **4** demonstrated strong anticancer activity against HeLa cells, which is intrinsic property of the Pt(II) corners **A1** and **A2** (Table 2). By light irradiation, however, the anticancer activity could be further amplified through inducing the photodynamic therapy modality, which is characteristic property of the BODIPY fluorophore, and have resulted in significant enhancement in the cytotoxicity against the cisplatin resistant cell line A2780cis. Thereby, effective theranostic agents have been obtained through combination of the chemotherapeutic potential of the platinum acceptors with the BODIPY donors as imaging probes and photosensitizers.

**Table 2:** Cytotoxicity data of selected metallacycles against a panel of human cancer cells.

**Compound**	**IC_**50**_ (hrs of incubation)/Type of cells (IC_**50**_ of ref. compound)**	**IC_**50**_ (hrs of incubation)/Type of cells (IC_**50**_ of ref. compound)**	**References**
**A1**	3.60 ± 0.21 μM (24 h)/HeLa (0.81 ± 0.08/CDDP)		Zhou et al., 2018a
**A2**	1.76 ± 0.14 μM (24 h) HeLa (0.81 ± 0.08/CDDP)	14.2 μM (24 h)/A2780cis (12.9/CDDP)	Zhou et al., 2018a
**3**	2.11 ± 0.17 μM (24 h) HeLa (0.81 ± 0.08/CDDP)	18.1 μM (24 h)/A2780cis (12.9/CDDP)	Zhou et al., 2018a
**4**	6.41 ± 0.38 μM (24 h) HeLa (0.81 ± 0.08/CDDP)		Zhou et al., 2018a
**3** **+** **light**	0.37 μM (*+24 h)/HeLa	0.76 μM (*+24 h)/A2780cis (12.9/CDDP)	Zhou et al., 2018a
**4** **+** **light**	0.95 μM (*+24 h)/HeLa		Zhou et al., 2018a
**BODIPY** **+** **light**	4.88 μM (*+24 h)/HeLa	6.09 μM (*+24 h)/A2780cis (12.9/CDDP)	Zhou et al., 2018a
**5a**	4.96 ± 0.17 μM (48 h)/U87 (6.06 ± 0.12/CDDP)	16.71 ± 0.33 μM (48 h)/**WI-38** (77.25 ± 7.28/CDDP)	Gupta et al., 2017
**5b**	3.43 ± 0.09 μM (48 h)/U87 (6.06 ± 0.12/CDDP)	9.16 ± 0.40 μM (48 h)/**WI-38** (77.25 ± 7.28/CDDP)	Gupta et al., 2017
**5c**	3.73 ± 0.02 μM (48 h)/U87 (6.06 ± 0.12/CDDP)	8.01 ± 0.19 μM (48 h)/**WI-38** (77.25 ± 7.28/CDDP)	Gupta et al., 2017
**5d**	6.39 ± 0.28 μM (48 h)/U87 (6.06 ± 0.12/CDDP)	15.54 ± 0.22 μM (48 h)/**WI-38** (77.25 ± 7.28/CDDP)	Gupta et al., 2017

*Cell types given in bold are normal non-cancerous cells*.

* *Irradiation by light (400–700 nm, 50 mW/cm^2^) for 5 min after 12 h incubation followed by12 h incubation*.

In another work, BODIPY derived Pd(II) metallosupramolecular triangles have been obtained by using Pd(II) hinges that are *cis*-capped with 1,3-bis-diphenylphosphino-propane, and the triangle-to-square conversion was studied (**5a-d** in Figure 1) (Gupta et al., 2017). The structures were fully characterized by ^1^H, ^31^P NMR, ESI-MS, and X-ray diffraction (of the triangle). The triangle and square topologies exist in a solvent-dependent equilibrium with the triangles being favored in higher polarity solvents. The stability of the complexes has been evaluated by UV-Vis absorption in presence of chlorides and in phosphate-buffered solution in DMSO or Dulbecco's Modified Eagle Medium, showing no change in the spectra for up to 48 h, except for the partial disruption seen upon addition of chloride ions. These BODIPY derived Pd(II) supramolecular triangles showed selective cytotoxicity against brain cancer (glioblastoma) cells U87 in comparison to that against normal brain cells WI-38. The anticancer activity of the triangles is higher or comparable to that of cisplatin, whereas the estimated selectivity is less pronounced than the selectivity of cisplatin (Table 2). These green fluorescent metallacycles have also been tested for fluorescence imaging of the cancer cells; the data demonstrated internalization in the cytoplasm and accumulation of aggregates in the cell membrane but not in the nucleus. Unlike the smaller precursors, the supramolecular triangles show appreciable interaction with the tested biomolecules—dsDNA, ssDNA, and BSA.

**Figure 2:**
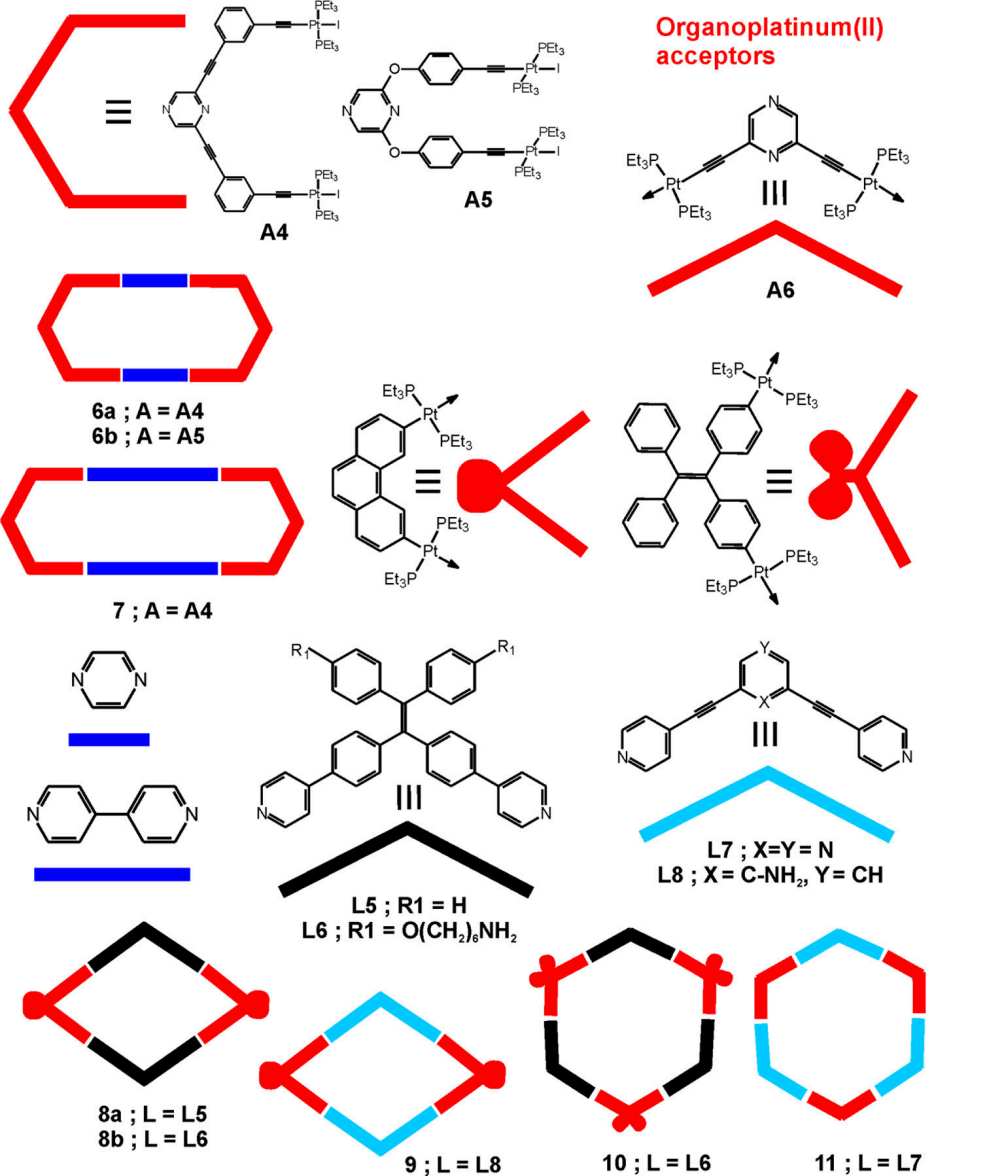
Metallacycles built from organoplatinum(II) acceptors and bent shaped ligands.

Hexagonal metallamacrocycles have been obtained by self-assembly of an organometallic platinum(II) clip **A4** with linear dipodal ligands, 4,4′-bipyridine or pyrazine, depicted in Figure 2 (Bhowmick et al., 2018). Both macrocycles (**6a** and **7**) exhibited cytotoxicity higher than cisplatin against the human breast cancer cell line MCF7. In general, the smaller-size macrocycle **6a** showed higher anticancer activity against all cell types (Table 3), and induced higher cell membrane damage. The latter has been associated with eventual necrosis of the cancer cells, in addition to the apoptosis, which was related to the swollen morphology of the treated cells seen by fluorescence microscopy imaging. The difference in the observed anticancer activity of the studied macrocycles has been associated with the lower hydrophobicity and lower molecular weight of **6a**; stability in presence of biomolecules has not been tested. Similarly to the macrocycles **6a** and **7**, the synthesis and anticancer potency of other flexible [2 + 2] tetracationic metallamacrocycles have been estimated (Jana et al., 2018). The macrocycle **6b** exhibited moderate anticancer activity against the tested cell lines (Table 3) as a result of self-assembly of the non-toxic organometallic clip **A5** and the pyrazine ligand. Yet, its cytotoxicity is lower than cisplatin and no selectivity could be attained.

**Table 3:** Cytotoxicity data of selected metallacycles against a panel of human cancer cells.

**Com-pound**	**IC_**50**_ (hrs of incubation)/Type of cells (IC_**50**_ of ref. compound)**	**IC_**50**_ (hrs of incubation)/Type of cells (IC_**50**_ of ref. compound)**	**IC_**50**_ (hrs of incubation)/Type of cells (IC_**50**_ of ref. compound)**	**IC_**50**_ (hrs of incubation)/Type of cells (IC_**50**_ of ref. compound)**	**References**
**6a**	20.0 ± 0.2 μM(48 h)/A549 (25.0 ± 0.3/CDDP)	05.0 ± 0.2 μM(48 h)/MCF7 (20.0 ± 0.3/CDDP)	16.0 ± 0.4 μM(48 h)/KB (8.0 ± 0.4/CDDP)	16.0 ± 0.5 μM(48 h)/HaCaT (12.0 ± 0.3/CDDP)	Bhowmick et al., 2018
**6b**	19.4 ± 0.5 μM HT-29 (1-10/CDDP)	9.1 ± 0.1 μM MCF-7 (1-10/CDDP)	9.6 ± 0.9 μM MDA-MB-231 (1-10/CDDP)	10.4 ± 0.2 μM**RC-124** (1-10/CDDP)	Jana et al., 2018
**7**	>30.0 μM (48 h)/A549 (25.0 ± 0.3/CDDP)	17.0 ± 0.3 μM(48 h)/MCF7 (20.0 ± 0.3/CDDP)	20.0 ± 0.1 μM(48 h)/KB (8.0 ± 0.4/CDDP)	20.0 ± 0.1 μM(48 h)/HaCaT (12.0 ± 0.3/CDDP)	Bhowmick et al., 2018
**A6**	21.54 ± 3.2 μM (48 h)/A549 (22.38 ± 2.9 /CDDP)	14.28 ± 1.2 μM(48 h)/HepG2 (18.89 ± 2.1 /CDDP)	19.55 ± 2.5 μM(48 h)/HeLa (10.21 ± 1.9/CDDP)		Jana et al., 2019
**11**	10.22 ± 1.6 μM (48 h)/A549 (22.38 ± 2.9 /CDDP)	11.02 ± 1.3 μM(48 h)/HepG2 (18.89 ± 2.1/CDDP)	8.73 ± 1.0 μM(48 h)/HeLa (10.21 ± 1.9/CDDP)		Jana et al., 2019
**13**	5.2 ± 2.0 μM/T98G (69.6 ± 7.9 /CDDP)	15.5 ± 2.3 μM/KB (72.6 ± 6.2 /CDDP)	18.3 ± 3.0 μM/SNU80 (49.3 ± 5.4 /CDDP)	36.2 ± 5.1 μM/**HEK293** (17.3 ± 3.6 /CDDP)	Mishra et al., 2014
**14**	4.5 ± 2.1 μM/T98G (69.6 ± 7.9 /CDDP)	13.0 ± 1.2 μM/KB (72.6 ± 6.2 /CDDP)	12.0 ± 2.8 μM/SNU80 (49.3 ± 5.4 /CDDP)	35.0 ± 3.8 μM/**HEK293** (17.3 ± 3.6 /CDDP)	Mishra et al., 2014

*Cell types given in bold are normal non-cancerous cells*.

Recently, two supramolecular metallacycles with hexagonal and rhombic shape have been obtained by using pyrazine-based organoplatinum(II) clip **A6** and two isomeric, also pyrazine-based, ditopic pyridyl ligands (Jana et al., 2019). The *para*- isomer of the ligand **L7** is shown in Figure 2 and leads to formation of the hexagonal metallacycle **11** upon [3+3] self-assembly with the pyrazine-based organoplatinum(II) acceptor, whereas the *meta*- isomer of **L7** forms a rhomboidal structure (not shown) upon [2+2] self-assembly with the same organoplatinum(II) acceptor. Both types of metallacycles showed slightly higher anticancer activity than the organoplatinum(II) acceptor **A6** and cisplatin (Table 3), which is more appreciable for the larger, hexacationic [3+3] self-assembly **11**.

**Figure 3:**
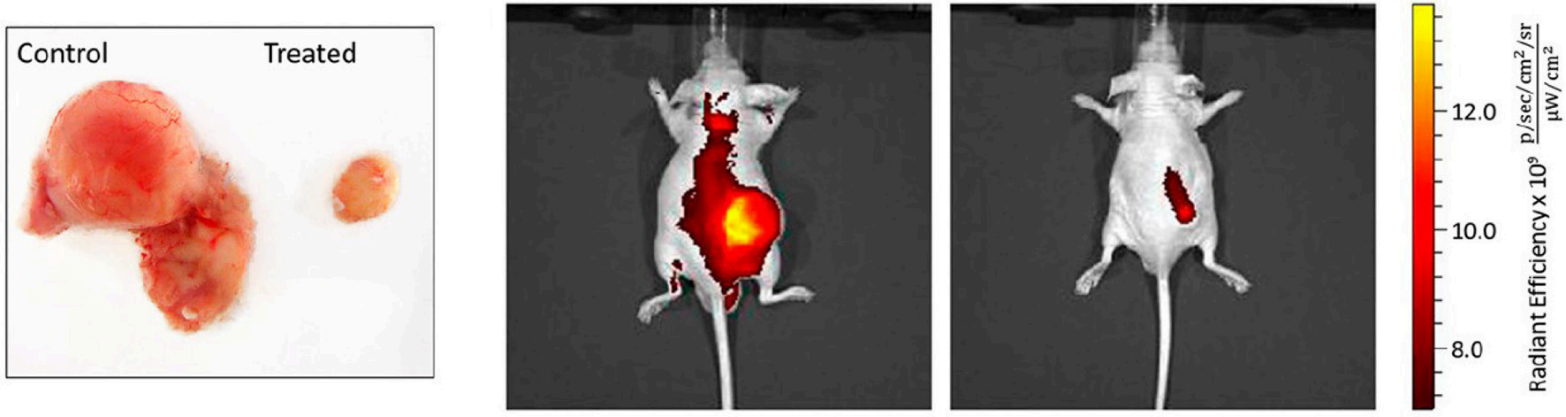
Effect of rhomboid **9** on tumor growth rate in MDA-MB-231 xenografts. **(Left)** Representative pictures of tumors, excised from control and mice treated with rhomboid **9**. **(Right)** Localization of the near-infrared contrast agent IR-783 in the tumors of the control and treated mice. The signal was processed with Living Image software with one representative sample for each group presented above. Mice from the rhomboid **9**-treated group show lower intensity of the signal originating from the tumor-accumulated contrast agent compared with the control group. Adapted with permission from Grishagin et al. (2014).

The rhomboidal Pt(II)-metallacycle **9**, depicted in Figure 2, has been evaluated for its anticancer activity both *in vivo* and *in vitro* (Grishagin et al., 2014). The formed metallacycle shows 13 times higher emission in the visible region (400–700 nm) than the parent ligand in aqueous-DMSO solution. This property has been used to monitor the cellular uptake and the stability of the metallacycle by laser-scanning confocal microscopy in A549 and HeLa cells. The data evidenced their stability upon cellular internalization in addition to the high resistance to photobleaching under the conditions of the confocal microscopy experiment. At low concentrations (1 nM−5 μM) the rhomboidal metallacycle and the organoplatinum(II) acceptor are not toxic, as seen by MTT test after 48 h of treatment of the cancerous cells. However, in the *in vivo* experiments in immunocompromised nude mice with tumor xenografts of breast cancer MDA-MB-231, the rhomboid **9** exerted a median tumor volume reduction of 88% (*P* < 0.001) (Figure 3). A maximum dose of 6 mg/kg in PBS:DMSO = 1:1 (v/v) has been applied by i.p. injections of 300 μL per animal, leading to no obvious signs of toxicity in terms of weight loss and visual appearance and behavior of the treated mice. The tumor growth inhibition (T/C%) value, defined as the ratio of the median tumor volume for the treated vs. the control group at a particular day, has been estimated to 36% at the last day of the experiment of 30 days (Figure 3 left).

**Figure 4:**
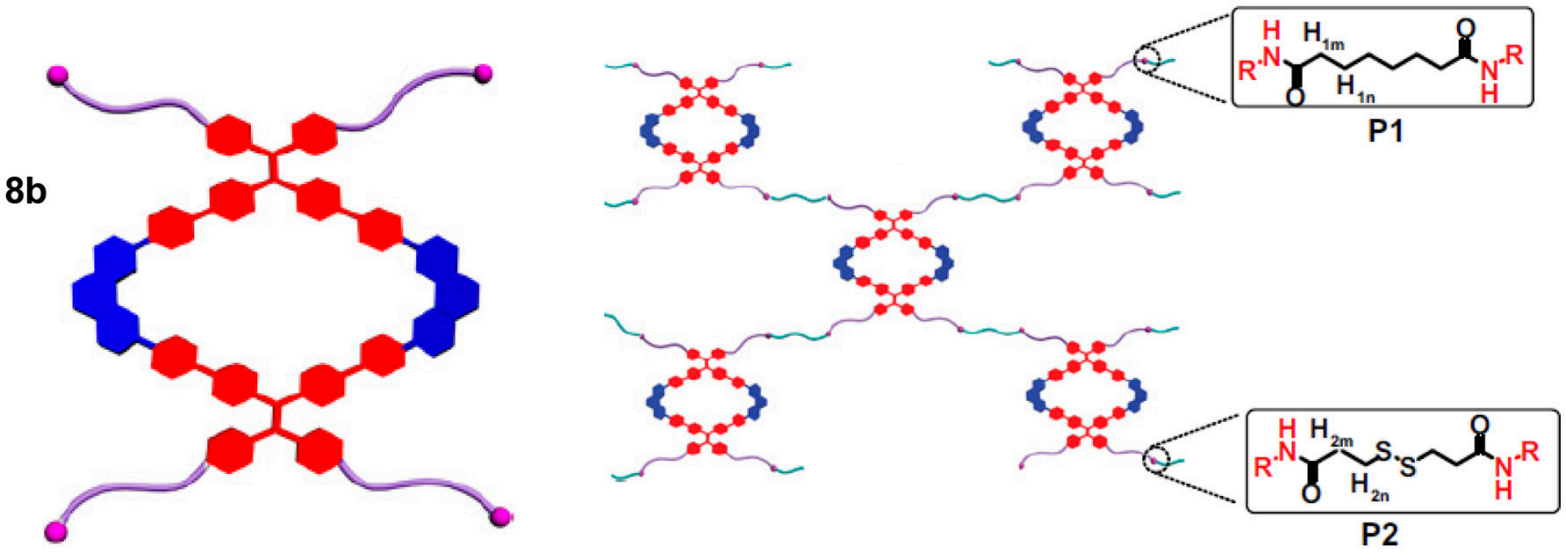
Polymeric networks in P1 and P2 built by interconnecting metallacylces **8b**. Adapted with permission from Zhang et al. (2016).

Stang and coworkers prepared metallosupramolecular polymers based on an organometallic Pt-linked rhomboidal metallacycle **8b** (Figure 2) that was conjugated with two types of polymers via amidation reaction between alkylamine and N-hydroxysuccinimide-activated carboxylic acid (Zhang et al., 2016). These metallacycle-cored polymers P1 and P2 (Figure 4) have been utilized to form nanoparticles at low concentration. All synthetic modifications resulted in enhanced emission due to aggregation-induced emission properties of the tetraphenylethene-based pyridyl donor that is the main building block of the metallacycle. The fluorescence properties of these polymers were further used in cell imaging, which showed a significant enrichment in lung cells after i.v. injection into a mouse bearing an MDA-MB-231 tumor. The observed significant fluorescence of the tumor even 24 h after injection demonstrated that the studied metallosupramolecular polymer (P2) is both chemostable and photostable *in vivo*. Considering the anticancer activity of previously reported rhomboidal Pt(II) metallacycles of Grishagin et al. (2014), the authors infer that this type of fluorescent metallacycle-cored polymers can have potential applications in lung cancer theranostics.

The aggregation-induced emission could also be exploited in building metallosupramolecular species as has been described for a hexagonal [3+3] self-assembled metallacycle **10** built from tetraphenylethylene (TPE)-based organoplatinum(II) acceptors and the ditopic TPE-based dipyridyl ligand **L6** (Figure 2), as well as that of octacationic cages that were obtained from a tetrapodal tetrapyridyl ligand similar to **L6** (Yan et al., 2016). Interestingly, both types of supramolecular assemblies showed distinct photophysical properties depending on the degree of flexibility that the ligand attains after self-assembling with the TPE-based platinum(II) acceptors. The hexacationic metallacycle **10** exhibits aggregation-induced emission (AIE) by increasing the amount of hexane in the CH_2_Cl_2_ solutions (up to 90%) due to the formation of nanoaggregates, as seen from the scanning electron microscopy (SEM) images, whereas the cages are intrinsically highly emissive. Taking advantage of the observed aggregation-induced enhanced emission (AIEE) of these systems, the same authors applied advanced approaches to build hybrid metallosupramolecular polymeric and bio-nanoparticles. The hexacationic metallacycle **10** has been assembled with the negatively charged rod-like tobacco mosaic virus (TMV) to form three-dimensional micrometer-sized superstructures via electrostatic interactions (Tian et al., 2016). In this way the fluorescence dramatically increased and provided an easily detectable evidence for the formation of novel metallosupramolecular biohybrid materials. Interestingly, addition of tetrabutylammonium bromide disrupts the metallosupramolecular core which results in lower fluorescence. The same strategy has been used to build similar biohybrid materials with bacteriophage M13 and ferritin, although with different degree of aggregation-induced emission. The demonstrated fluorescence switching upon aggregation with negatively charged protein-based species, forming emissive bionanoparticle/metallosupramolecular hybrid complexes, and their controllable disruption, enrich significantly the possibilities for design and development of different supramolecular assemblies with dynamic optical properties and high potential to be used for both imaging and delivery of bioactive compounds.

**Figure 5:**
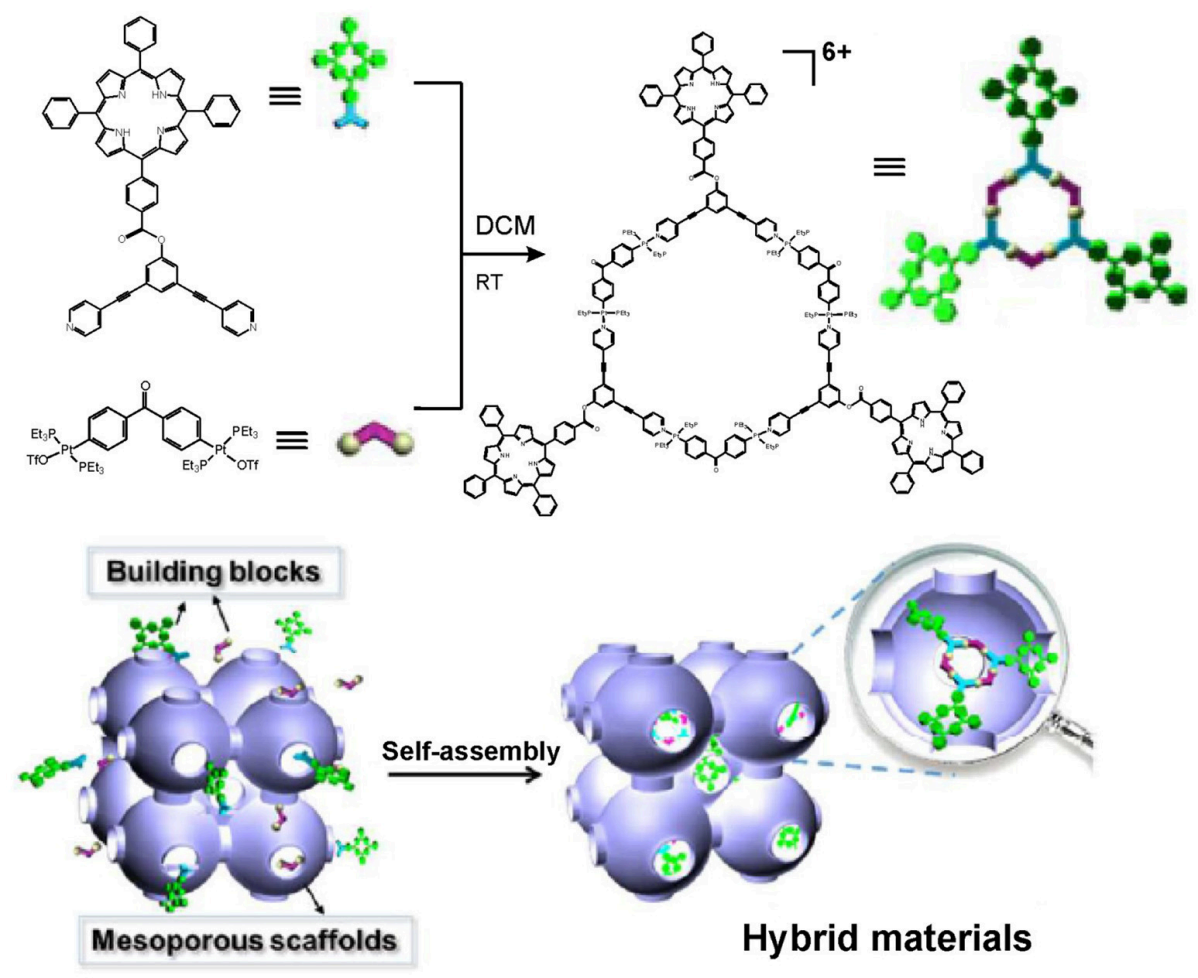
Construction of porphyrin-containing metallacycle within the confined cavity of mesoporous carbon FDU-16. Adapted with permission from Chen et al. (2018); Copyright 2018 American Chemical Society.

On the other hand, aggregation-induced quenching may significantly compromise the photophysical functionality of some fluorophores such as porphyrins. In a recent paper, an alternative way to produce porphyrin functionalized hexagonal metallacycle has been reported (Chen et al., 2018). To avoid self-aggregation of the porphyrin rings, the metallacycle depicted in Figure 5 has been produced in the confined cavity of mesoporous carbon FDU-16. Thereby, the molecules of the porphyrin-containing metallacycle have been dispersed within the mesoporous cavities, which resulted in increased stability and photocatalytic activity of the porphyrin-containing metallacycles. These properties have been demonstrated through successful use of the hybrid material in heterogeneous catalysis for photooxidation of sulfides. The six-fold faster generation of singlet (^1^O_2_), compared to that of free metallacycles in solution, is an important improvement that has been achieved. Although no biological applications have been discussed yet, the demonstrated strategy to construct well-defined porphyrin-containing metallacycles with improved stability and photoactivity may find implications in the future development of nanohybrids with improved properties for PDT.

**Figure 6:**
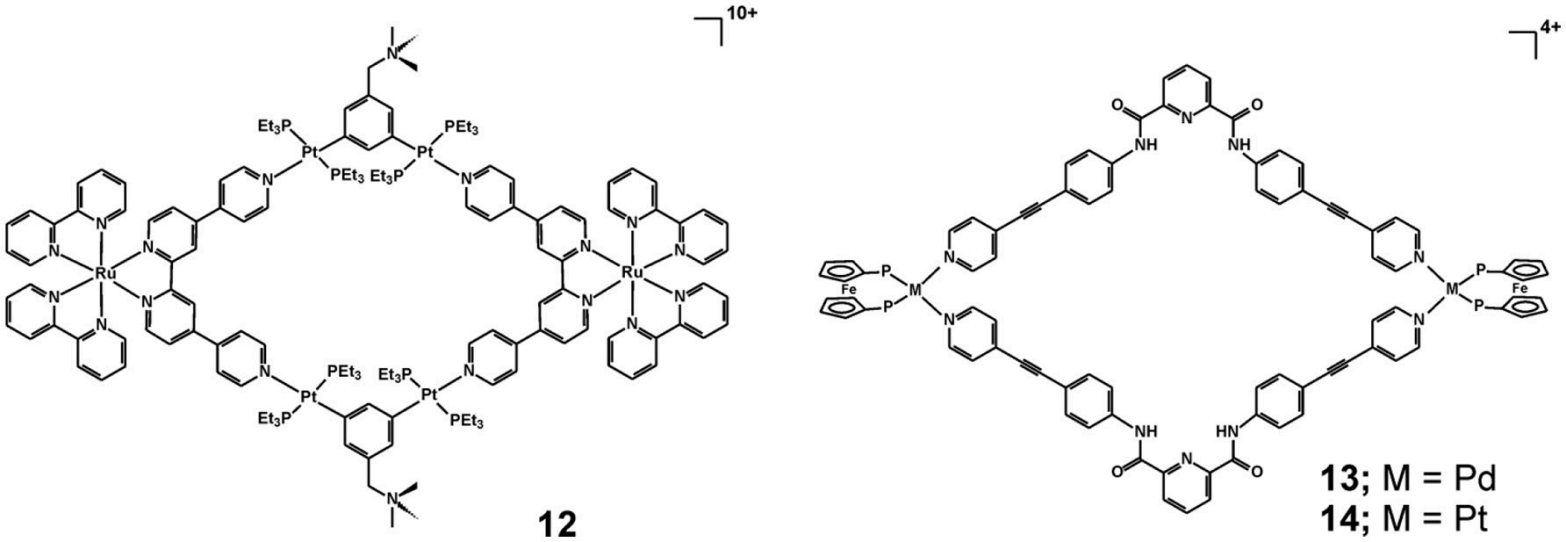
Heterobimetallic cycles **12** – **14** with notable optical, electrochemical, and anticancer properties.

Another strategy to build metallosupramolecules with significantly augmented photophysical properties, which allow for their use in photodynamic therapy, has recently been demonstrated for a heterometallic Ru-Pt metallacycle **12** (Figure 6) (Zhou et al., 2018b). The authors utilized the excellent photostability and two-photon absorption characteristics of the Ru(II) polypyridyl precursor, which after formation of the metallacycle exhibits shifted luminescence to the near-infrared region characterized also with a larger two-photon absorption cross-section and higher singlet oxygen generation efficiency. Thereby, the formed large macrocyclic structure of **12** renders it a very potent two-photon PDT agent. It is proposed that the highly charged (decacationic) structure of **12** facilitates its internalization in cells and localization in mitochondria and nuclei as evidenced by time-dependent inductively coupled plasma MS (ICP-MS) analysis of cellular uptake and the biodistribution of **12** in A549 cells. The metallacycle exhibits very high photocytotoxicity, induced by the generation of intracellular ^1^O_2_ upon irradiation and damaging the mitochondria and cellular nuclei, whereas the toxicity in the dark is very weak. These observations have also been confirmed in the *in vivo* studies which demonstrated that **12** is capable to efficiently ablate cancer tissues at low light doses with minimal system toxicity.

The tetracationic heterobimetallic cycles **13** and **14** have been obtained from an N, N′-bis(4-(pyridin-4-ylethynyl)phenyl)pyridine-2,6-dicarboxamide ligand and *cis*-blocked palladium(II) or platinum(II) 1,1′-bis(diphenylphosphino)ferrocene triflates (Mishra et al., 2014). Their structure and composition have been characterized by multinuclear NMR spectroscopy, elemental analysis, and high-resolution electrospray mass spectrometry (HR-ESMS) and further modeled by molecular mechanics calculations. The stability of the metallacycles has been tested either by monitoring the cytotoxicity effect after different periods of pre-incubation in cell culture medium (Dulbecco's modified eagle medium—DMEM) or in DMSO (for 0, 10, 25, and 50 h at 37 ^0^C) or by fluorescence spectroscopy measurements in physiological buffer solution at pH 7.4 and in presence of redox active compounds (H_2_O_2_ or dithiothreitol). While both metallacycles exhibit cytotoxicity higher than cisplatin against the tested human-cancer cell lines (T98G, KB and SNU-80), they are twice less toxic to the normal human embryonic kidney cells (HEK-293) than cisplatin, which points to a much better cancer cell selectivity (Table 3). The stability studies indicated that under physiological conditions and even in presence of redox-active compounds the metallacycles **13** and **14** remain intact for at least 50 h, whereas in presence of DMSO a significant loss of cytotoxicity has been seen for pre-incubation periods of > 25 hours. These observations could suggest that the metallosupramolecular structure of **13** and **14** is crucial for the observed cytotoxicity profiles, since the DMSO induced disassembly is proposed as the main reason for decrease in the cytotoxicity. The mode of action leading to the observed cytotoxicity has been associated with apoptosis as evidenced by the caspase-3/7 activity assays.

## Metallacages and Helicates

Metallacages and helicates are 3D structures that have characteristic cavity or cylindrical shape. Thereby, except combining the properties of the building components, metallacages can be excellent hosts for a variety of guest molecules that can be encapsulated in the hollow of the cages. This particular feature of metallacages has long been utilized in the design and development of smart nanostructures for multiple applications, such as molecular flasks (Yoshizawa et al., 2009), containers (Zarra et al., 2015), for safe storage (Yamashina et al., 2014) and recognition (Rodriguez et al., 2017), etc. Metallahelicates, on the other hand, can have specific helicity that is capable of selective interactions with biomolecules with certain conformation, such as DNA fragments. The growing interest in exploring the biomedical applications of these types of 3D metallosupramolecules has progressed to building metallacages that are either capable of encapsulating bioactive molecules or can be structurally decorated with biofunctional fragments. Thereby, the areas of potential applications of metallacages already include drug delivery and bioimaging except the initially explored biological activities that are intrinsically characteristics for their components. Similar expansion in the exploration of the properties of metallahelicates is also in progress.

**Figure 7:**
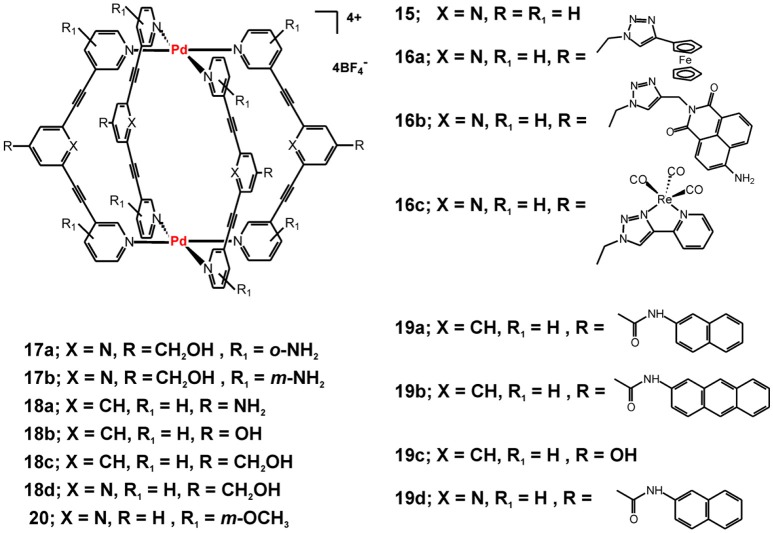
Metallosupramolecular cages with general formula [Pd_2_L_4_](BF_4_)_4_ built from a series of tri- or bi-pyridyl ditopic ligands.

Some examples of metallacages that had attracted the attention of synthetic and bioinorganic chemists are the [Pd_2_L_4_]^4+^ cages that assemble from simple ditopic tripyridyl ligands−2,6-bis(pyridin-3-ylethynyl)pyridine or its bis(pyridin-3-ylethynyl)benzene analog (Figure 7). The interest has been initiated with the reported encapsulation of the anticancer drug cisplatin by the supramolecular cage **15** that can be controllably released by external stimuli (Lewis et al., 2012). The cage formation and cisplatin encapsulation proceeds virtually quantitatively and the cage **15** and its host-guest complex with encapsulated two molecules of cisplatin have been characterized by single-crystal X-ray diffraction analyses. While the cage and the host-guest complex are rather stable in organic solvents, the cage readily disassembles in presence of competing amine ligands or chlorides, and thus the encapsulated cisplatin molecules can be released. The authors studied in details the encapsulation phenomenon and highlighted the importance of the hydrogen bonding interactions between the cage and the amine ligands of the cisplatin guest. This conclusion has been suggested by the lack of encapsulation evidences when a Pd_2_L_4_ metallacage built from 1,3-bis(pyridin-3-ylethynyl)benzene ligand (when X = CH in **15**, Figure 7) has been used.

Further exploration of the observed stimuli-responsive catch and release of cisplatin by cage **15** has been focused on suitable ligand modifications to include an optically responsive fragment and/or increase the stability of the cage and the host-guest complex under biologically relevant conditions. The Cu(I)-catalyzed azide-alkyne cycloaddition “click” reaction (CuAAC) has been utilized to build a library of electrochemically or photochemically responsive cages (**16a-c**, Figure 7) (Lewis et al., 2013, 2014). It has been demonstrated that the formed 1,2,3-triazole units in the exo-functionalization does not affect the self-assembling process nor does it interfere with the ability of the formed cages to bind cisplatin as guest molecules. Similarly, single-crystal X-ray structure has been determined for the 1:2—host-guest complex of cage **16a** and cisplatin (Lewis et al., 2013). The cage formation does not alter the one-electron reversible oxidation characteristic for 4-ferrocenyl triazoles. The same strategy has been used to “click” photoresponsive or biological molecules, such as 4-amino-1,8-naphthalimide (in **16b**, Figure 7) and fac-[Re(CO)_3_] complex with the 4-(2-pyridyl)-1,2,3-triazolyl moiety (in **16c**, Figure 7) or caffeine, acetate protected-D-glucose, estradiol and dipeptide units (Lewis et al., 2014). The attachment of the organic or inorganic fluorophores (in **16b** and **16c**, respectively) transformed the virtually non-emissive cages into emissive ones, with especially efficient emission seen for cage **16b** showing quantum yield of fluorescence of 0.59.

With the ultimate goal to improve the kinetic stability of the studied Pd_2_L_4_ cages and evaluate their biological activity, Crowley and coworkers further modified the used tripyridyl ligands by adding NH_2_-susbstituents in the terminal pyridyl rings (Preston et al., 2016). Thereby, cages **17a** and **17b** have been obtained and their kinetic and biological properties have been compared with the non-functionalized cage **18d**. The stability studies showed that 2-amino substitution on the terminal pyridine rings significantly enhances the stability of the formed cage **17a** in presence of chloride ions or histidine and cysteine—potent ligands in biological environment. Interestingly, cage **17b** has kinetic properties and cisplatin-encapsulation ability similar to that of **18d**, which points out to the importance of subtle structural modification of the self-assembling ligands on the properties of the formed metallacages. Enhanced stability of **17a** of up to several hours to almost 2 days (in presence of histidine) reflected also to the observed appreciable cytotoxicity against the cisplatin-resistant breast cancer cell line MDA-MB-231 (Table 4). In contrast, the **17b** and **18d** did not show appreciable cytotoxicity against the tested lung and breast cancer cell (for 24 h of incubation), which can be in favor to their stimuli-responsive cisplatin encapsulation and the suggested drug-delivery potential.

**Table 4:** Cytotoxicity data of selected metallacages against a panel of human cancer cells (Figure 7).

**Compound**	**IC_**50**_ (hrs of incubation)/Type of cells (IC_**50**_ of ref. compound)**	**IC_**50**_ (hrs of incubation)/Type of cells (IC_**50**_ of ref. compound)**	**IC_**50**_(hrs of incubation)/Type of cells (IC_**50**_ of ref. compound)**	**References**
**15**	41.4 ± 3.9μM (24 h)/A549 (9.4 ± 0.3/CDDP)	56.7 ± 2.2 μM(24 h)/MDA-MB-231 (41.2 ± 3.9/CDDP)	70.1 ± 13.8 μM 24 h)/DU145	McNeill et al., 2015
**17a**	50 μM (24 h)/A549 (9.4 ± 0.3/CDDP)	36.4 ± 1.9 μM(24 h)/MDA-MB-231 (41.2 ± 3.9/CDDP)		Preston et al., 2016
**18a**	16.5 ± 4.3 μM (72 h)/A549 (8.9 ± 4.2/CDDP)	16.7 ± 2.0 μM(72 h)/SKOV-3 (15.4 ± 2.2/CDDP)	8.2 ± 1.6 μM (72 h)/HepG2 (6.4 ± 1.5/CDDP)	Schmidt et al., 2016b
**18b**	47.3 ± 1.8 μM (72 h)/A549 (8.9 ± 4.2/CDDP)	66.0 ± 10.8 μM(72 h)/SKOV-3 (15.4 ± 2.2/CDDP)		Schmidt et al., 2016b
**18c**	13.2 ± 2.4 μM (72 h)/A549 (8.9 ± 4.2/CDDP)	11.6 ± 1.7 μM(72 h)/SKOV-3 (15.4 ± 2.2/CDDP)	10.7 ± 0.6 μM (72 h)/HepG2 (6.4 ± 1.5/CDDP)	Schmidt et al., 2016b
**18d**	32.0 ± 9.7 μM (72 h)/A549 (8.9 ± 4.2/CDDP)	44.0 ± 6.2 μM(72 h)/SKOV-3 (15.4 ± 2.2/CDDP)	29.7 ± 2.6 μM (72 h)/HepG2 (6.4 ± 1.5/CDDP)	Schmidt et al., 2016b
**19a**	5.9 ± 1.4 μM (72 h)/A549 (8.9 ± 4.2/CDDP)	8.0 ± 1.4 μM(72 h)/SKOV-3 (15.4 ± 2.2/CDDP)		Schmidt et al., 2016a
**19b**	1.1 ± 0.3 μM (72 h)/A549 (8.9 ± 4.2/CDDP)	1.1 ± 0.6 μM(72 h)/SKOV-3 (15.4 ± 2.2/CDDP)		Schmidt et al., 2016a
**19c**	82.6 ± 15.1 μM (72 h)/A549 (8.9 ± 4.2/CDDP)	94.4 ± 7.9 μM(72 h)/SKOV-3 (15.4 ± 2.2/CDDP)		Schmidt et al., 2016a
**19d**	1.4 ± 0.5 μM (72 h)/A549 (8.9 ± 4.2/CDDP)	1.2 ± 0.7 μM(72 h)/SKOV-3 (15.4 ± 2.2/CDDP)		Schmidt et al., 2016a
**20**	71.8 ± 9.1 μM (48 h)/A549 (16.8 ± 0.7/CDDP)	44 ± 11 μM(48 h)/HepG2 (6.7 ± 0.9/CDDP)		Kaiser et al., 2016
**21-hex**	6.9 ± 0.9 μM (24 h)/A549 (9.4 ± 0.3/CDDP)	6.0 ± 0.6 μM(24 h)/MDA-MB-231 (41.2 ± 3.9/CDDP)	3.4 ± 0.4 μM (24 h)/DU145	McNeill et al., 2015
**22**	5.1 μM/HBL100 (4.9/CDDP)	6.7 μM/T47D(28.3/CDDP)		Hotze et al., 2006
**23**	0.16 μM/HBL100 (4.9 μM/CDDP)	0.29 μM/T47D(28.3 μM/CDDP)		Hotze et al., 2006
**24**	22 μM/HBL100 (4.9 μM/CDDP)	53 μM/T47D(28.3 μM/CDDP)		Pascu et al., 2007

Further functionalization and cytotoxicity studies on this class of metallacages have been undertaken by Casini, Kühn and coworkers. The antiproliferative effects of Pd(II) cages of hydroxymethyl-functionalized bi- and tripyridyl ligands (**18c** and **18d** in Figure 7, respectively) indicated that upon 72 h of incubation the cages exert cytotoxicity against the tested cancer cells either comparable to that of cisplatin (for **18c**) or 4-times lower (for **18d**, Table 4) (Schmidt et al., 2016b). The other two examples of Pd(II) cages derived from differently functionalized bipyridyne ligands (**18a** and **18b** in Figure 7) have the same order of cytotoxicity effect as **18c**. The observed lower toxicity of **18d** and its distinctive ability to encapsulate cisplatin, as suggested earlier by Crowley and confirmed herein by X-ray data of the host-guest complex, can be regarded as favorable combination of properties for the sake of anticancer drug delivery potential (Schmidt et al., 2016b). To introduce optical functionality to the observed higher cytotoxicity of the Pd(II) cages derived from the bi-pyridyl ligands, the same authors exo-functionalized the Pd_2_L_4_ cages with naphthalene or anthracene fluorophores (Schmidt et al., 2016a). The formed ligands exhibit weak fluorescence and upon self-assembly and formation of the cages **19a** – **19d** (Figure 7) the emissive properties of the fluorophore-tagged ligands have been preserved leading to the formation of weakly or non-emissive metallacages, most probably due to the high degree of flexibility. The exo-functionalization with aromatic rings, however, reflected the cytotoxicity of the ligands and the corresponding metallacages **19a, 19b**, and **19d** that increased considerably when compared with the non-functionalized cage **19c** (Table 4). Interestingly, by introducing methoxy groups in the terminal pyridine rings the fluorescence of the ligands increases (Kaiser et al., 2016); upon capsule formation, however, virtually non-emissive Pd(II)- and a novel Pt(II) cage have been obtained. Interaction of the Pd(II) cages of metoxy-functionalized bi- and tri-pyridine ligands with cisplatin has been suggested by ^1^H NMR measurements. Pd(II) cage **20** and its analog of a bi-pyridine ligand (structure not shown) have been proposed as suitable drug-delivery platforms as the estimated cytotoxicity is relatively low.

To achieve efficient targeting, functionalization of metallacages with biological fragments has been reported for the Pd_2_L_4_ cages of the di-pyridyl ligands depicted in Figure 7 (Han et al., 2017). Two different approaches for exo-functionalization with a model linear peptide Ac-NLEFK-Am have been described, which are based on amide bond formation between the carboxylic (or amine) group of the exo-functionalized ligand/cage and that of the side chains of the model peptide. Better results could be obtained with the pre-conjugation approach, where the peptide is coupled to the ligand prior to the following *in situ* self-assembly and formation of the Pd_2_L_4_ cages. Thereby, metallacages of larger size could be obtained that can more efficiently benefit from the enhance permeability effect (EPR) for passive targeting to cancer cells and tissues. More importantly, this approach could open the prospect to bioconjugation and specific targeting of metallosupramolecules by attaching receptor-specific peptides or antibodies as an way to complement their either drug-delivery potential or intrinsic cytotoxicity (Han et al., 2017).

**Figure 8:**
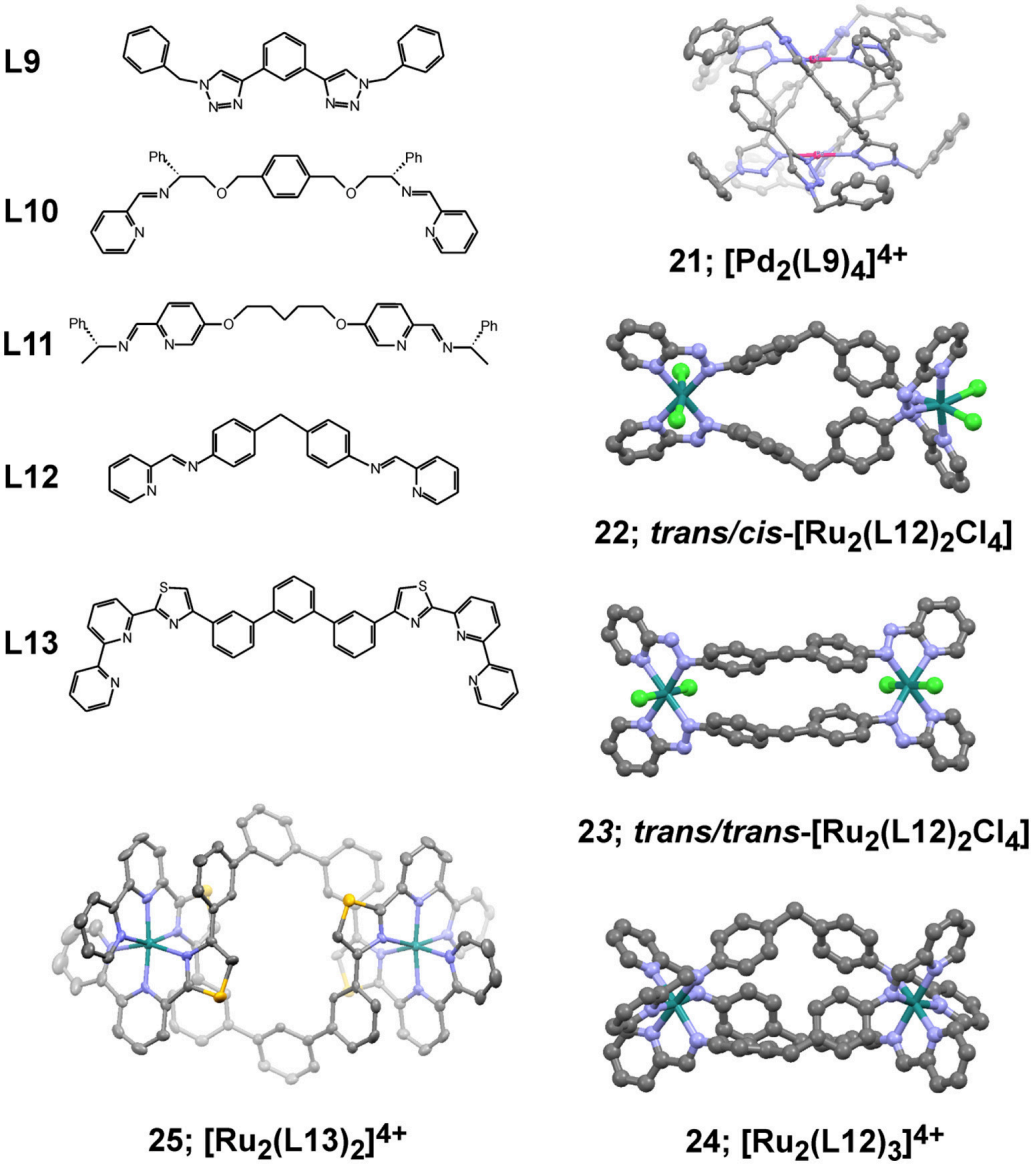
Dipodal ligands (**L9** – **L13**) for building helical metallosupramolecules similarly to represented crystallographic views of selected examples (**21** – **25**). Views of the crystal structures are produced with Mercury3.8 software from the corresponding files: **21** – CCDC 762916; **22** – CCDC 603391; **23** – CCDC 603392; **24** – CCDC 636261; **25** – CCDC 1566897; all are freely available from www.ccdc.cam.ac.uk.

Metallosupramolecular systems of composition [Pd_2_L_4_]^4+^ have been isolated by using smaller size ditopic ligands based on bis-triazoles similar to **L9** in Figure 8 (McNeill et al., 2015). The formed complexes have the form of quadruply-stranded helicates similarly to the crystal structure of **21**, depicted in Figure 8 and reported earlier (Crowley and Gavey, 2010). These helical structures have improved stability that is much more pronounced in the case of bis-triazole ligand with hexyl pendant arms forming the analogous complex **21-hex** (structure not shown). The increased stability in presence of chlorides, histidine and cysteine correlated with the observed higher cytotoxicity against the tested cancer cell lines in comparison of the metallacage **15** (Table 4). Interestingly, the complex **21-hex** attains its half-inhibitory concentration the first 2 h of treatment of the cells in contrast to the known anticancer platinum drugs. This suggested different mode of anticancer action that has been associated with loss of cellular membrane integrity as derived from fluorescence microscope imaging and enzymatic (LDH) activity assay (McNeill et al., 2015). This hypothesis also corroborated with observed lack of selectivity of the cytotoxicity of complex **21-hex**. A large library of bis(bidentate) 2-pyridyl-1,2,3-triazole ligands with various lengths have been explored for their ability to form triple helicates with Fe(II) ions with general composition [Fe_2_L_3_]^4+^ (Vellas et al., 2013). While self-assembly and formations of cylinders have been confirmed in solution as well as in the solid state, the poor stability of these complexes hampered their potential biological activity. Molecular docking studies of these complexes suggested they could bind duplex and triplex DNA fragments.

The chiral metallohelical complexes [Fe_2_(**L10**)_3_]^4+^ and [Fe_2_(**L11**)_3_]^4+^, constructed by Scott, Qu and coworkers, have shown evidences to selectively target and enantioselectively inhibit amyloid-β (Aβ) aggregation (Li et al., 2014). The ability of these metal complexes to inhibit Aβ assembly suggested that they might be useful in blocking Aβ-mediated cellular toxicity and thereby present a therapeutic strategy for treatment of Alzheimer's disease. The selective targeting of alpha/beta-discordant stretches at the early steps of aggregation has been demonstrated by multiple biophysical and biochemical approaches. More recently, a series of analogs of [Fe_2_(**L11**)_3_]^4+^, having linking bridges of different flexibility, have been tested for their anticancer activity and evidenced both very good water solubility and stability (Kaner et al., 2016). Interestingly, the so-called flexicates exhibit anticancer activity of sub-micromolar to nanomolar range against the tested human cancer cell lines: MDA-MB-468 (human epithelial breast adenocarcinoma), HCT116 p53^+/+^ and HCT116 p53^−/−^ that are genetically identical human colorectal cancer cell lines except for the presence or absence of functional p53, which is a common genetic difference associated with increased resistance to chemotherapy. In the cisplatin-sensitive cells MDA-MB-468, the glycolbridged flexicate analogous to [Fe_2_(**L11**)_3_]^4+^ have shown a cytotoxicity (IC_50_ = 0.2 ± 0.1 μM) higher than cisplatin by an order of magnitude. Moreover, some of the tested flexicates were very toxic against the resistant HCT116 p53^−/−^cells that lack p53 and showing cytotoxicity as low as 40 nM. Another important feature of this class of flexicates is their excellent selectivity, which is estimated to be nearly three orders of magnitude higher than that of cisplatin against healthy human retinal pigment epithelial (ARPE19) and lung fibroblast (WI38) cells. The helical complex [Fe_2_(**L10**)_3_]^4+^ showed limited solubility in water and could not be fully characterized, whereas its *m*-xylenyl analog exhibited cytotoxicity as high as 0.4 ± 0.1 μM against the MDA-MB-468 cell lines. In some cases significant enantiomeric difference had been observed pointing to the structural and conformational importance of the helicates. Nevertheless, the investigated mode of action could not be associated with DNA interaction nor apoptosis induced cell death.

The earlier reported metallosupramolecular helicate [Fe_2_(**L12**)_3_]^4+^ of Hannon and coworkers are capable of binding DNA Y-shaped junctions and could also unwind DNA and bind preferentially to regular alternating purine–pyrimidine sequences (Malina et al., 2008). More recent studies on the DNA interaction preferences have also revealed that this metallohelicate is capable of recognizing DNA bulges that are DNA defects consisting of one or more unpaired bases in the DNA double helical strands (Malina et al., 2014). These findings complemented the available data on the ability of the [Fe_2_(**L12**)_3_]^4+^ helicate to recognize non-canonical structures in DNA that might have relevance with various diseases. Recent report on the ability of the [Fe_2_(**L12**)_3_]^4+^ helicate to bind to TAR RNA, which is transactivation responsive region RNA, suggested it biological potential of inhibiting an essential step in the HIV-1 replication cycle (Malina et al., 2016). The observation that the iron(II) supramolecular helicates binds to TAR RNA at nanomolar concentrations and inhibits the interaction between the HIV-1 transactivator protein Tat and TAR, which plays a critical role in HIV-1 transcription, broadens the scope of biological importance of metallosupramolecular helicates.

The anticancer potency of dinuclear ruthenium metallohelicates have been among the first examples of biological activity demonstrated for double-stranded (**22** and **23** in Figure 8) (Hotze et al., 2006) and triple-stranded (**24** in Figure 8) metallohelicates (Pascu et al., 2007). The two different isomers of the dinuclear [Ru_2_L_2_Cl_4_] complex exhibit slightly different cytotoxicity profiles (Table 4) with the symmetric **23** isomer being an order of magnitude more cytotoxic than the cis/trans isomer **22** against the tested cancer cell lines. The analogous triple-stranded helicate **24** (Pascu et al., 2007) demonstrated very high stability and capability of binding DNA. This cylinder is photoresponsive to additions of ct-DNA in terms of fluorescence enhancement from the ruthenium cylinder and a concomitant blue shift (8 nm) in the emission maximum. The observed cytotoxicity, however, is significantly lower than the double-stranded analogs and also lower than that of cisplatin (Table 4). More recent examples on the construction and biological activity of double-stranded Ru(II) helicates of **L13** have been reported by Rice and coworkers and suggested the formed helicate **25** as a suitable compound that can target difficult to treat tumors (Allison et al., 2018), such as the human colorectal cancer cell line HCT116. The selectivity of **25** was similar to cisplatin and oxaliplatin toward the HCT116 p53^+/+^ cancer cells, but it also showed substantively higher cytotoxicity toward the p53^−/−^ cells.

**Figure 9:**
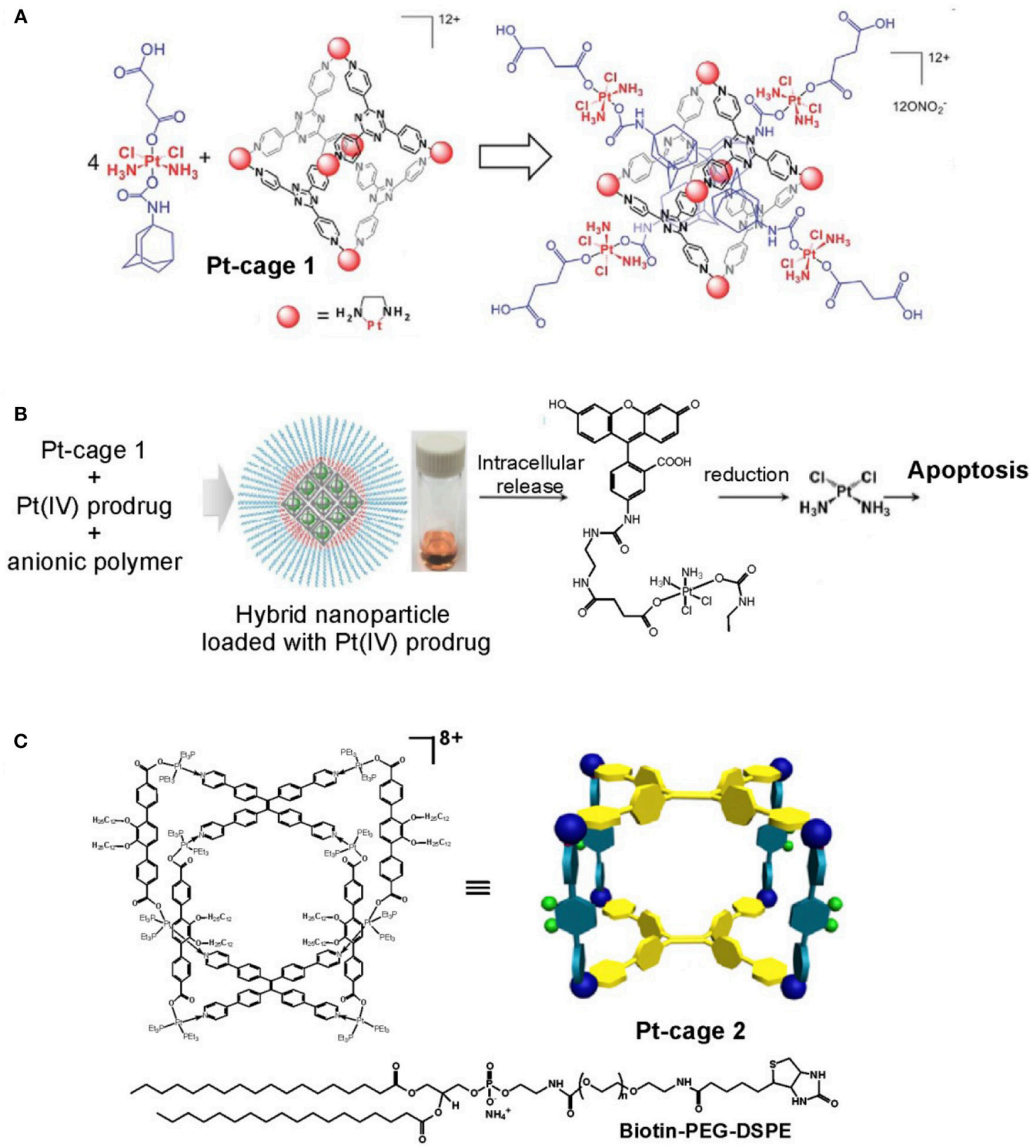
Advanced metallo supramolecular systems constructed from platinum metallacages. **(A)** Pt(IV) adamantyl prodrug encapsulated in the rhombic **Pt-cage 1** (adapted from Zheng et al., 2015; Published by The Royal Society of Chemistry); **(B)** hybrid metallosupramolecular-polymeric nanoparticles built from host-guest complex of fluorescein-conjugated Pt(IV) prodrug and **Pt-cage 1** coated with anionic polymer shell (adapted from Yue et al., 2018; with permission from RSC); **(C)** theranostic supramolecular nanoparticles built from highly emissive metallacage, **Pt-cage 2**, and biotin-conjugated PEGylated surfactant (adapted from Yu et al., 2016).

Advanced approaches that utilize metallosupramolecular cages as delivery system for either anticancer drugs and prodrugs or fluorescent agents have gained increasing interest as alternative ways to achieve selectivity and efficient targeting of tumor tissues for both therapeutic and imaging purposes. Among the initial examples are those that use the hexanuclear octahedral metallacage of Fujita (**Pt-cage 1** in Figure 9), (Ibukuro et al., 1998), which has been shown to encapsulate four molecules of adamantyl Pt(IV)-complex exhibiting prodrug properties (Zheng et al., 2015). The formed host-guest complex has a diameter of about 3 nm, as characterized by detailed NMR analyses. The cytotoxicity of the host-guest complex is much higher than these of the **Pt-cage 1** or the Pt(IV)-prodrug guest alone, and is comparable to the anticancer activity of cisplatin against a panel of human cancer cells lines. The improved anticancer activity is caused by biological reductions of the Pt(IV)-prodrug, as evidenced by adding ascorbic acid, and release of the cytotoxic cisplatin. Thereby the cisplatin resistance in the tested A280CP70 cell lines could be overcome that was explained with the high cellular uptake of the highly positive charge of the host-guest nanostructure. On the other hand, by changing the type of the Pt(II)-cornering complexes of **Pt-cage 1**, from ethilenediamine to an 1,1′-bipyridine complex, the formed octahedral cage becomes more cytotoxic than cisplatin against a panel of human cancer cells including the cisplatin-resistant celss (IC_50_ = 3.42 ± 0.63 μM for A2780CP70 and IC_50_ = 7.85 ± 0.21 μM for MD-MBA-231 vs. IC_50_ values for cisplatin of 6.49 ± 1.40 μM for A2780CP70 and 15.4 ± 2.3 μM for MDA-MB-231) (Zheng et al., 2016). The detailed study on the mechanism of action of this Pt_6_L_4_ octahedral cage showed that it interacts with DNA in a non-covalent intercalative way leading to DNA damage, and induces apoptosis through upregulation of p53 and p21 proteins.

Another strategy has been tested in building drug-loaded nanoparticles with polymeric shell of anionic block copolymer (methoxy polyethylene glycol-blockpolyglutamic acid, MPEG5k-PGA30) (Yue et al., 2018). The same octahedral metallacage (**Pt-cage 1**) has been used to encapsulate a fluorescein-linked Pt(IV) prodrug (Figure 9B). The Pt(IV) prodrug-loaded supramolecular nanoparticles have spherical shape, and their average size was estimated to 20 nm by cryo-TEM imaging or overall size of 80 nm including the peripheral polymers and according to the dynamic light scattering (DLS) data. The drug-release profiles speeds up dramatically under acidic conditions (pH = 5.0 in acetate buffer) and reducing environment of 1 mM ascorbic acid, that is estimated by the release half-life being 3 days in PBS and only 23 h in acidic and reducing environment. Intracellular release of the fluorescein-conjugated Pt(IV) prodrug was evidenced by fluorescence microscopy that has been enabled by the appearance of bright green fluorescence of the prodrug released from the otherwise non-fluorescent nanoparticle. Moreover, the cytotoxicity of the nanoparticles against HeLa cells is comparable to that of cisplatin (IC_50_ = 5.02 ± 0.67 μM for the nanoparticles; 2.95 ± 0.42 μM for cisplatin, and 5.98 ± 0.58 μM for the Pt(IV) prodrug), whereas the metallacage (**Pt-cage 1**) is essentially non-toxic. This work demonstrates an working strategy to use anionic block copolymer, to include a cytotoxic host-guest complex into negatively charged nanoparticles of appropriate size that are able to slowly release the therapeutic content and show excellent anticancer efficacy accompanied with fluorescence imaging modality.

Alternatively, Stang and coworkers utilized a highly emissive tetraphenylethene metallacage to build supramolecular nanoparticles with 1,2-distearoyl-phosphatidylethanolamine (DSPE)/polyethylene glycol (PEG) shell (Figure 9C) showing excellent stability and biotin-receptor targeting ability to be used as cancer-cell selective delivery systems (Yu et al., 2016). Moreover, these supramolecular nanoparticles preserve the coordination triggered aggregation induced emission (AIE) enhancement, which renders them highly emissive in biological media and suitable for imaging purposes. The incorporated metallacage is built from Pt(II) linked triphenyl pillars, decorated with differently modified PEG-arms, and two tetraphenyl-ethene ligands each bearing four pyridines for coordination with the metal. The metal precursor is a *cis*-Pt(Et_3_P)_2_(OTf)_2_ complex that exhibits high and unselective cytotoxicity. Upon formation of self-assembled polymeric aggregates with sizes of about 37 nm (as seen by TEM and DLS techniques) selective delivery to cancer cells has been achieved employing the EPR effect additionally to the biotin-mediated internalization in cancer cells. Thereby, the metallacage loaded nanoparticles combine the therapeutic with the imaging modality making them suitable for theranostic applications. Confocal laser scanning microscopy and flow cytometry confirmed the selective targeting of the biotin functionalized nanoparticles to cancer cells overexpressing the corresponding receptor (HeLa and HepG2 cells) in contrast to normal cells (CHO and HEK-293), which also corroborated with their higher and selective cytotoxicity and high Pt-content in the cancer cells nuclei. The theranostic functions of the biotin-functionalized nanoparticles have been demonstrated also *in vivo* in HeLa tumor bearing female nude mice confirming their superiority than the clinical formulations of oxaliplatin, carboplatin, or cisplatin used as control. Importantly, while higher accumulation of Pt from the nanoparticles was observed in the tumor in comparison with the control Pt-drugs, administration of nanoparticles resulted in a lower Pt uptake by the liver, spleen, lung, and kidneys, which suggested that the use of PEGylated nanoparticles also provides way to decrease the systemic toxicity of Pt-drugs.

## Metallocapsules and Barrels

**Figure 10:**
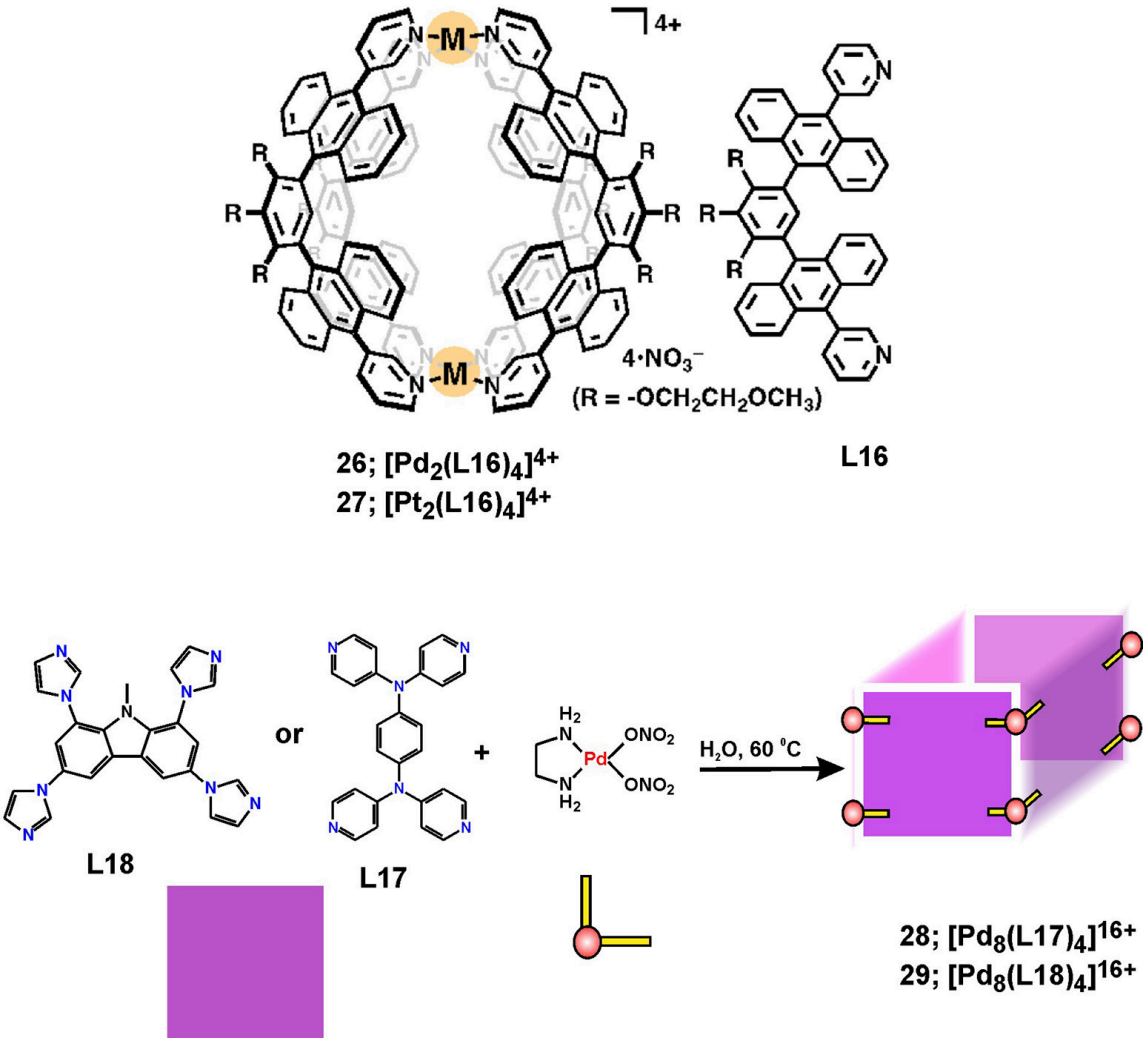
Metallosupramolecular capsules **26** and **27** and barrels **28** and **29** with characteristic hydrophobic cavities.

The design and utilization of large aromatic panels as multidentate donor ligands enabled the construction of 3D metallosupramolecular structures with a characteristic well-confined cavity, and having capsular or barrel-like shapes. Interesting examples consist of aromatic panels with intrinsic fluorescence properties, such as anthracene and carbazole fragments in the ligands depicted in Figure 10. The formation of robust M_2_L_4_ coordination capsules have been described by Yoshizawa and coworkers (Li et al., 2012), and their fluorescence and guest-encapsulation properties have been studied in details (Kishi et al., 2011) for the sake of practical applications for safe storage of reactive compounds (Yamashina et al., 2014) or recognition purposes (Yamashina et al., 2016, 2017).

Recent results on the anticancer properties of Pd(II) and Pt(II) capsules **26** and **27** (Figure 10) indicated that they exhibit anticancer activity profiles much superior to cisplatin in terms of higher toxicity against two types of leukemic cells (HL-60 and SKW-3) as well as against cisplatin-resistant cells (HL-60/CDDP) (Ahmedova et al., 2016b). More importantly, the capsules showed more than 5 times better selectivity toward cancerous cells than that of cisplatin, which has been estimated by the IC_50_ of normal-to-cancer cell toxicity ratio employing human embryonic kidney cells (HEK-293) as a model for non-malignant cells. The stability tests revealed that both types of coordination capsules remain intact in presence of excess of biologically relevant molecules such as amino acids and DNA bases. However, the addition of sulfur-containing biomolecules such as cysteine, causes disassembly of the capsules that proceeds instantaneously in case of the Pd(II) capsule **26** and takes up to 3 days for the more inert Pt(II) analog **27**. Decomposition of the capsules can be easily monitored by the appearance of the characteristic strong fluorescence of the liberated bis-anthracene ligand **L16**. Further on, the effect of guest encapsulation on the stability and the cytotoxicity of the capsules have been investigated (Ahmedova et al., 2016a). A successful modulation of these properties has been achieved by encapsulation of different types of hydrophobic guest molecules (pyrene and caffeine). The stabilities of the empty hosts and the corresponding host-guest complexes have been tested in presence of glutathione – the sulfur-containing oligopeptide that has relevance to cancer evolution. The observed strong stabilization of the capsules by encapsulation of pyrene guests has resulted in virtually complete loss of cytotoxicity of the otherwise highly toxic empty hosts **26** and **27** against the tested human cancer cell lines (HT-29, T-24, HL-60 and its resistant counterparts HL-60/Dox and HL-60/CDDP). These findings have suggested that the cytotoxicity of **26** and **27** can be readily modulated by encapsulation of large aromatic guests. More interestingly, the observed cytotoxicity profiles correlated with the capsules stability in the presence of glutathione that was estimated by NMR-based kinetic experiments. The observed stability-cytotoxicity correlation allowed proposing that the glutathione-triggered decomposition of the capsules could be the reason for the observed anticancer activity of these robust capsular metallosupramolecules. This hypothesis corroborates with the reported very high selectivity of the capsules to cancerous cells as well as with their cross-resistance cytotoxicity.

Mukherjee and coworkers reported the synthesis and single-crystal X-ray structure of a Pd_8_L_4_ water-soluble molecular barrel **28** that self-assembled from a tetrapyridyl donor (**L17**) and a cis-blocked Pd(II) precursor [cis-(en)Pd(NO_3_)_2_] (Bhat et al., 2017). The affinity of the barrel's cavity to hydrophobic guest molecules has been employed in encapsulation of the anticancer drug curcumin, which resulted in significant stabilization of curcumin and its solubilization in water. This reflected on the increased anticancer activity of water solutions of curcumin against HeLa cancer cells demonstrating the successful utility of the metallosupramolecular barrel as a carrier and protector for photosensitive and hydrophobic drugs. Although stability and cytotoxicity of the empty barrels alone have not been investigated, this study clearly demonstrated the increased bioavailability and stability of curcumin in water solutions as seen from the fluorescence microscopy imaging of the cancer cells and the increased cytotoxicity with respect to the curcumin concentration.

A similar tetrafacial barrel with composition Pd_8_L_4_ (**29**, Figure 10) has been built from tetraimidazolyl carbazole ligand (**L18**) (Roy et al., 2015). This metallosupramolecule has a tubular morphology that was fully characterized by multinuclear 1D/2D NMR and ESI-MS as well as by single-crystal X-ray diffraction of the coronene enclosing host-guest complex. While the barrel itself is water soluble, its hydrophobic cavity renders it ideal to solubilize hydrophobic guest molecules. Moreover, the inclusion of polyaromatic hydrocarbons with strong optical absorption and emission properties is accompanied with color and/or emission change. This property has been employed in live-cell fluorescence microscopy imaging by the water soluble perylene enclosing barrel, which demonstrated enhanced cell membrane permeability of the inclusion complex. This example gives the hope for further successful utilization of water soluble metalosupramolecules as drug or probe carriers provided their biocompatibility, stability and drug release properties is proved.

The flexible tetra(pyridin-4-yl)benzene-1,4-amine (**L17**, Figure 10) has also been used to construct organometallic octanuclear Ru(II) cages from dinuclear p-cymene ruthenium(II) acceptors, used as bridging oxo-pillars that vary from oxalate, benzoquinones, naphthoquinones (Ajibola Adeyemo et al., 2017). The largest box was characterized by X-ray diffraction besides the NMR, ESI-MS and electronic absorption spectra that have been used for characterization of all other boxes. All Ru8 cages showed strong anticancer activity *in vitro* that is comparable with cytotoxicity of cisplatin against A549 and HeLa cells. The anticancer profiles showed increasing cytotoxicity by enlarging the aromatic unit of the oxo-bridging pillars.

## Conclusions

From the presented recent examples for the fast-expanding chemotherapeutic applications of metallosupramolecular entities, it can be concluded that this class of compounds provide a rich field for exploration. The diversity in their properties stems from the fact that the metallosupramolecules can intelligently combine metal hinges of different coordination and reactivity properties as well as multidentate ligands that can be readily decorated without affecting the self-assembly processes. This has presented several working strategies to either increase the size or stability of the formed metalosupramolecular structures or to link them with receptor specific vectors. Construction of larger size composites that incorporate metallosupramolecules proved useful to target and deliver both therapeutic and imaging probes. The unique property of the 3D structures of metallacages, capsules, and barrels, is their discrete cavity which opens up further prospects for biological applications. Among the mostly explored ones are the encapsulation and delivery of bioactive molecules. Thereby, one and the same metallosupramolecular host can be used for different biological (therapeutic or diagnostic) purposes depending on the properties of the encapsulated guest molecule. The demonstrated encapsulation of Pt(IV)-prodrugs, which can be activated and subsequently released in the intracellular media by selective reduction and formation of the corresponding Pt(II)-drug, is an intriguing advancement to fully explore the potential for biomedical applications of the coordination supramolecules. Moreover, the encapsulation of a fluorescein-bearing Pt(IV)-prodrugs by the **Pt-cage 1**, shown in Figure 9, had actually added imaging modality to the selective anticancer treatment. The presented examples are only some of the suggestions on how to augment the cancer treatment potential of such host-guest complexes with additional imaging modality.

The showed examples can expand in the near future toward modifying or multiplying the main therapeutic action by introducing immunomodulatory, anti- or pro-oxidative molecules, or other bioactive fragments either as guest molecules or as exo-functionalization of the supramolecular hosts. The proof-of-concept for exo-functionalization of metallosupramolecules with large peptides has already been demonstrated and is expected to open additional prospect to construct hybrid systems with larger size (>30 nm) that can benefit from both the targeting properties of the attached biomolecule and the truly efficient EPR effect.

The flexibility to modulate the overall stability of the 3D metallosupramolecular structures, by simply encapsulating appropriate guest molecule is another approach to fine-tune either the delivery properties or their therapeutic potential. In the cases when the increased stability affects the biological action of the supramolecules, guest-encapsulation can be exploited to turn the toxic species into non-toxic or vice versa, and thus, transform them from toxic anticancer agents to safe drug-delivery vehicles. Exploiting also building components with interesting optical or redox properties enriches further the possibilities to adjust the structure and properties of the constructed metallosupramolecules to the desired biomedical application. Inevitably, the presented examples are an expanding source of inspiration for many synthetic and biological chemists to, hopefully, allow them fully reveal the multifaceted potential of the metallosupramolecules.

## Author Contributions

The author confirms being the sole contributor of this work and has approved it for publication.

### Conflict of Interest Statement

The author declares that the research was conducted in the absence of any commercial or financial relationships that could be construed as a potential conflict of interest.

## Abbreviations

CDDP: cisplatin
SISO: cervix adenocarcinoma
A-427: lung carcinoma
LCLC-103H: large cell lung cancer
5637: urinary bladder carcinoma
U2OS: osteosarcoma
VM-1: cisplatin resistant melanoma cell line
MCF7: breast cancer
A549: lung carcinoma
KB: oral cancer
HaCaT: skin keratinocyte
MDA-MB-231: human breast adenocarcinoma cell line
T98G: (brain tumor)
SNU-80: (thyroid cancer)
HEK-293: human embryonic kidney cells (non-malignant cell lines)
HepG2: human hepatocellular carcinoma
DU-145: prostate cancer cells
MCF10A: immortalized non-malignant breast tissue
SKOV-3: Human ovarian cancer
HBL100 and T47D: Human breast-tumor cell lines
HT-29: colorectal adenocarcinoma cells
T-24: bladder cancer cells
SKW-3: T-cell leukemia
HL-60: acute myelocyte leukemia
HL-60/Dox and HL-60/CDDP: Doxorubicin and cisplatin-resistant counterparts of the HL-60 cells.
